# KinImmerse: Macromolecular VR for NMR ensembles

**DOI:** 10.1186/1751-0473-4-3

**Published:** 2009-02-17

**Authors:** Jeremy N Block, David J Zielinski, Vincent B Chen, Ian W Davis, E Claire Vinson, Rachael Brady, Jane S Richardson, David C Richardson

**Affiliations:** 1 Biochemistry Department, Duke University Medical Center, Durham, NC 27710, USA; 2 Visualization Technology Group, Pratt School of Engineering, Duke University, Durham, NC 27706, USA; 3 Electrical and Computer Engineering Department, Pratt School of Engineering, Duke University, Durham, NC 27706, USA; 4 Biochemistry Department, University of Washington, Seattle, WA 98195, USA

## Abstract

**Background:**

In molecular applications, virtual reality (VR) and immersive virtual environments have generally been used and valued for the visual and interactive experience – to enhance intuition and communicate excitement – rather than as part of the actual research process. In contrast, this work develops a software infrastructure for research use and illustrates such use on a specific case.

**Methods:**

The Syzygy open-source toolkit for VR software was used to write the KinImmerse program, which translates the molecular capabilities of the kinemage graphics format into software for display and manipulation in the DiVE (Duke immersive Virtual Environment) or other VR system. KinImmerse is supported by the flexible display construction and editing features in the KiNG kinemage viewer and it implements new forms of user interaction in the DiVE.

**Results:**

In addition to molecular visualizations and navigation, KinImmerse provides a set of research tools for manipulation, identification, co-centering of multiple models, free-form 3D annotation, and output of results. The molecular research test case analyzes the local neighborhood around an individual atom within an ensemble of nuclear magnetic resonance (NMR) models, enabling immersive visual comparison of the local conformation with the local NMR experimental data, including target curves for residual dipolar couplings (RDCs).

**Conclusion:**

The promise of KinImmerse for production-level molecular research in the DiVE is shown by the locally co-centered RDC visualization developed there, which gave new insights now being pursued in wider data analysis.

## Background

3D molecular structures and their visualizations are central to the progress of biology and medicine in the last century, which has steadily followed our ability to observe finer and finer biomolecular detail and then integrate it to higher levels of structural complexity. Although the information-storage capability of DNA arises from its sequence and the specificity of its base pairing, almost all other biological functions (catalysis, gene expression, specific binding, cellular structure, growth, signaling, mobility, etc.) are due to the detailed 3D structural relationships between the atoms of protein and nucleic acid molecules. Knowing those structural relationships is essential to understanding our own, and all the rest of, biology.

### The Tools: Molecular graphics and virtual reality

Just as 3D structure is central to biomolecular function, 3D visualization is central to understanding those structures and functions. Macromolecular structure truly uses the three spatial dimensions and is inherently complex, cooperative, handed, irregular, and mobile in 3D. Even for communicating specific structural concepts, static 2D images are only second-best [1], while the discovery of new relationships is enormously enhanced in interactive systems that fully explore the third dimension. Since these molecules are smaller than the wavelength of light, there is no such thing as "photorealism" in their rendering. Therefore the emphasis is on varied and appropriate representations that provide the best insight and that can be tailored to suit the concepts being studied or conveyed.

In the beginning days of structural biology, interactive computer graphics was not possible for macromolecules, and comprehension of the structures came from physical models that were labor-intensive, expensive, and susceptible to time and gravity. The two most widely used types were the Kendrew skeletal models where the bonds are brass rods and the atoms are their joints (thus showing connectivity and identity well, and open enough to see and even reach into the center of a molecule, but not conveying volume and surface) and the Cory-Pauling-Koltun (CPK) space-filling models where each atom is a plastic sphere of slightly under van der Waals radius (colored by atom type and showing surface shape and atom interactions directly, but obscuring interior information and connectivity).

The earliest molecular graphics on the computer used Cα or all-atom "stick" representations, with a single drawn vector per bond allowing rotation on high-end machines of the time [2]. With the advent of bitmap displays, both the disadvantages and the advantages of CPK models were reproduced in static gray-scale computer images [3], with real-time performance possible only much later. Such vector and CPK systems enabled analysis of active sites and recognition of structural motifs. Interactive fitting of crystallographic models into electron density contours was first achieved with the Grip-75 system at UNC Chapel Hill [4], then adopted in Frodo [5], and became an essential part of determining protein crystal structures. With the advent of color, dot surfaces [6] became the first widely successful innovation in molecular representation to originate on the computer side. In combination with stick models, they allow the dual 1D connectivity and 3D positional nature of macromolecules to be visualized at the same time: bonded atomic interactions by the sticks and non-bonded surface interactions by the dots. Ribbon schematics developed as 2D hand drawings for publication [7] were adapted for 3D computer graphics in the 1980's [8].

In the early 90's molecular graphics migrated to personal desktops and into classrooms, with kinemages and Mage [9] or with RasMol [10]. Since then, faster hardware has enabled great improvements in the capabilities of such displays. Many excellent software systems are now available for interactive 3D molecular graphics, such as PyMol [11], DeepView [12], KiNG [13], Chimera [14], MolMol with specific NMR features [15], Coot with specific crystallographic features [16], and VMD with specific molecular-dynamics features [17]. The current state of the art on fast desktops or laptops – with flexible representations, high resolution, stereo, manipulation and calculation features, and smooth rotation for full models as big as the ribosome – is an enormously effective tool, widely cited and illustrated, in routine use by all structural biologists and biomedical researchers.

The most interactive and immersive of computer graphics are those that use virtual reality techniques, such as head-mount displays and trackers, force-feedback, tracked gloves or wands, multi-sense modalities, wall-size displays, and surround-projection CAVEs [18]. Such immersive virtual environments have made a great impact in many fields from gaming to surgery, but have so far seen only limited use for macromolecular structures, primarily as part of the VR demonstration repertoire.

Over the last thirty years the computer science department at UNC Chapel Hill has contributed to virtual reality systems for investigating molecules, including a head-mount display system, a two-wall display for joint molecular graphics work by two people [19], the first system for user "tugs" on a molecule with real-time energy minimization [20], and especially focusing on force-feedback "haptic" devices, which they have developed into an effective system for actual physical manipulation of molecules in conjunction with atomic-force microscopes [21]. This nanomanipulator is a case where a macromolecular virtual reality tool has become a standard part of the actual research process [22]; another such case is the Crumbs volume-rendering VR tool [23] which is used in biomedical imaging. However, both examples are at the lower-resolution level of microscopy, rather than the level of atomic detail addressed by the KinImmerse system.

The first multi-wall CAVE virtual environment was developed 15 years ago [24]. It was very early made to display molecules, by means of a plug-in for the VMD molecular-dynamics display software [17] using Sherman's FreeVR library [25]. Most users of the VMD software package are researchers who run molecular dynamics simulations and the sophisticated functions in VMD are geared towards those users, such as display of non-spatial variables across the simulation trajectory or user tugs on the calculated atom motions [26]. After submission of the current work, a paper was published [27] describing the MDDriver libraries that interface generic molecular dynamics (MD) software to a VR version of VMD, providing interactive user tugs with haptic feedback to guide the course of a simulation. It seems likely this could in future enhance MD research in a VR environment, but it does not provide the tools for general structural biology and structural bioinformatics research uses as aspired to here. The VMD-based systems support multi-processor VR, but not cluster-based VR such as the DiVE used in this project (see Methods).

Simpler, general-purpose macromolecular visualization systems for VR include Amira , a commerical software package which provides support for molecular viewing of PDB files in CAVE-type displays. A group at University of California Irvine modified MolScript on an SGI Onyx to display molecules on the 4-screen CAVE at Mississippi State [28]. That system can show and move ball&stick or ribbon representations of several proteins at once, floating above a gridded floor. A report in the Protein Data Bank newsletter [29] describes a system called PDB in a CAVE, built on the COVISE platform and providing VR displays of proteins and nucleic acids in ribbon and other representations from web-downloaded PDB files, including animation capability. So far it has been used interactively in demonstration mode with large wall displays, but is also capable of display in CAVEs. The Jean Goldwurm 3D Visualization Theater at the Weizmann Institute of Science [30] has a large wall display in stereo, supporting standard graphics software such as PyMol or Amira. It is intended for use by audiences of scientific collaborators as well as for educational purposes.

All of the above systems provide effective molecular visualization in some type of VR. However, they all use standard representations (usually ribbons, ball&stick, and CPKs); many are not open source and hence not modifiable for new uses; only Amira supports measurements; and only the VMD-based systems have any tools for model manipulation or other research interactions. In recent years, most of the early limitations to VR and especially CAVE systems (low resolution, slow rendering, tracking latency) have been overcome, creating the opportunity to try for production-level molecular research applications. The work reported here develops the KinImmerse open-source software with very flexible display creation and suitable interface tools for molecular-structure research within the 6-surface surround of the DiVE or other VR system, and explores a test application for NMR structures.

### The Application Area: Macromolecular structures and NMR ensembles

The 3D coordinates and experimental data for macromolecular structures are made publicly available in the international Protein Data Bank, or PDB [31], which currently contains over 50,000 entries. There are two principal experimental techniques for determining these structures in atomic detail: x-ray crystallography and NMR (Nuclear Magnetic Resonance spectroscopy). Both depend on constructing a molecular model consistent with many thousands of individual measurements and also with bond lengths and angles known from chemistry and with the amino acid or base sequence. The logic of the two methods differs, however – crystallographic data directly give position in 3D space but atom identities must be inferred, while NMR data show local distance or angle relationships between identified atoms but their positions must be inferred. NMR data and the resulting models therefore have an inherently local, relational perspective. The two types of NMR data central to 3D structure determination are: 1) the NOE (Nuclear Overhauser Effect) that measures through-space distance between two atoms closer than about 5Å, and 2) the RDC (Residual Dipolar Coupling) that measures an angular relationship between a specific interatomic bond vector and the tensor describing molecular orientation to the magnetic field in partially-ordered experimental samples [32]. The RDC value measured for a specific pair of atoms places that bond direction somewhere along a symmetrical pair of ellipse-like target curves (see Results). Crystal structures are typically reported as a single model, with a "B-factor" estimate of positional uncertainty. NMR structures are reported as ensembles of multiple models each of which is consistent with the data; differences between the models can result either from incomplete data or from real motion in the molecule [32].

Both crystal and NMR structures are very reliable, especially those with the most experimental data (high resolution for x-ray, many restraints per residue for NMR), but both are susceptible to occasional large local inaccuracies (often caused by systematic errors in data interpretation) that can hurt their uses for other biomedical research. We have contributed new methods for the diagnosis and correction of local misfittings in protein and RNA crystal structures [33,13,34], which have proven highly effective in routine use [35,36].

We would like to develop related methods suitable for improving the accuracy of NMR structures. That is a harder task because of the less direct relationship of the experimental data to 3D space, the complication of multiple models, and a tradition of determining and analyzing the models computationally rather than visually, and globally rather than locally. Good molecular graphics for NMR ensembles are especially challenging for those same reasons. So far, NMR graphics use neither display of paired RDC target curves on the model, nor an explicitly local, immersive context as suggested by the local nature of the experimental data (especially the exclusively short-range NOEs). We believe that RDC-curve display and immersive VR, with the user zoomed-in inside the molecular ensemble, should be particularly effective for utilizing that local perspective to better understand both the NMR methodology and the resulting structures.

## Methods

### VR hardware

**Figure 1:**
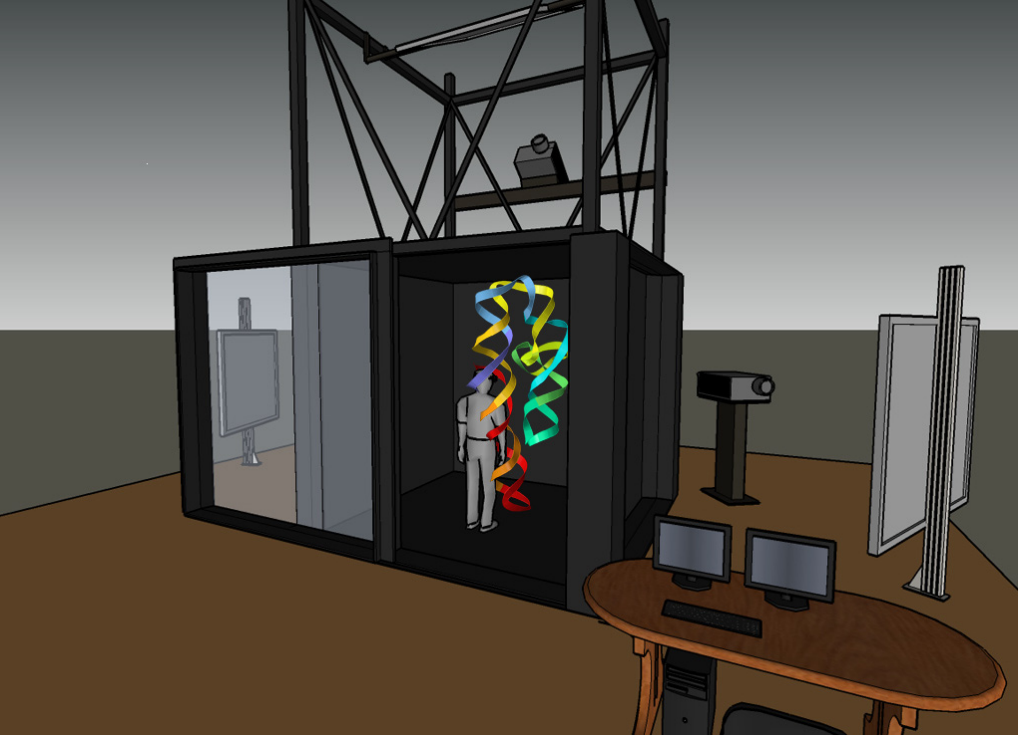
**The DiVE**. Schematic showing the physical configuration of the 6-sided Duke immersive Virtual Environment (DiVE), operated by the Visualization Technology Group . The sliding-door front wall is open, and two of the large stand mirrors are shown that direct images from the projectors onto the translucent walls; a sixth projector is beneath the floor.

These displays were shown in the Duke Immersive Virtual Environment (DiVE), a 6-sided, fully immersive VR system approximately 2.9 m × 2.9 m × 2.9 m, illustrated schematically in Figure 1. The walls are flexible black screen with wooden and acrylic frame, and can be removed for screen replacement. The ceiling and floor are rigid acrylic, 20 mm and 50 mm thick, respectively. The door (the entire front wall) opens manually by sliding.

Images on each of the six sides currently have 1056 × 1056 resolution, with stereo switched between eyes at 110 Hz. Each of the six images is shown by a 3000-lumen Christie Mirage S+2k DLP projector. The displays are calculated and distributed through a cluster of seven machines connected by 1 Gigabit Ethernet: one master node and a separate client node for each of the six projectors. Each node is a Sun W2100z running Windows XP, with dual 2-GHz processors, 2 Gb of memory, and an NVidia 3000 G graphics card.

Active stereoscopic vision is enabled through the CrystalEyes system from StereoGraphics Corp., including a master pair of stereo goggles that is head-tracked to set the the viewing point and direction. A hand-held 3D mouse or 'wand' from InterSense Technologies includes a joystick and four button controls. The InterSense IS-900 inertial/ultrasound tracking system determines the position and orientation of the stereo glasses and the hand-held 3D controller.

### Input of data

The molecular coordinate data for VR display in KinImmerse typically originates in files downloaded from the international Protein Data Bank [31]. The PDB-format file is parsed either on the MolProbity web site [34], or in the Prekin-Mage system [9,37], or in the KiNG kinemage display program [13], where molecular representations can be produced, modified, and then output in the kinemage graphics format [9], a hierarchical, human- or machine-readable text file format for the display of various graphics primitives. KiNG (in Java) and Prekin-Mage (in C) are open-source, multi-platform, and available from .

The first proof-of-concept system for displaying molecules in the DiVE was implemented using a modified output from KiNG and a Virtools (Dev 3.5; ) application that constructed VR displays from the KiNG output. That system showed the feasibility and promise of the kinemage-to-DiVE route (see Results), but required a commercial software system (Virtools) and had the limitation, traditional in VR, of using only surface-graphics primitives and not points or lines. For example, a covalent bond vector, which would be represented by a line in normal kinemage format, became a narrow 4-sided cylinder in the Virtools application; this decreases performance and hurts perception in crowded images. Both of the above limitations are overcome in the present KinImmerse program, which uses the open-source Syzygy toolkit and implements line as well as surface graphics objects. In the Virtools application, an all-atom ball&stick representation of a 400-residue protein updated at only 15–25 frames/second, which is slow enough to spoil the illusion of reality. In the current KinImmerse version, either a ribbon or a stick figure of the 50S ribosome (3000 residues of RNA and 3700 residues of protein) updates at 40–55 frames/second, more than adequate to swing it around smoothly. KinImmerse reads kinemage files directly, and the current version has implemented recognition of a large portion of the kinemage format (such as the group/subgroup/list hierarchy, pointID information, colors, line widths, ribbons, etc.). Input to KinImmerse is thus a kinemage-format file, either a pre-existing one or one created to suit the current VR objectives.

### The Syzygy toolkit

In order to support a variety of immersive virtual reality systems, the software programming toolkit "Syzygy" [38] was utilized for developing KinImmerse. It provides an abstracted interface to the programmer so that regardless of the particular display system, tracking system, operating system (support for Linux, MSWindows, MacOSX), or number of networked render nodes used, the application itself does not have to be modified. By instead using XML configuration files, Syzygy provides display support for head-mounted displays, cave-type systems, or tiled display walls, and also a desktop simulator mode useful for development. In order to facilitate the best immersive experience, a head and hand tracking system is often utilized. Syzygy directly supports a number of tracking systems, as well as many more through its interfaces to VRPN and Trackd. VRPN is a public domain library which provides a device-independent and network-transparent interface to virtual-reality peripherals [39]. Trackd is a commercial library which "takes information from a variety of tracking and input devices and makes that information available for other applications to use" [40].

### Callback-style API

The Syzygy toolkit is easy to use, since its interface is somewhat similar to the GLUT API application programming interface [41], which is familiar to most programmers in the OpenGL community. In order to convey what work was necessary to achieve the final KinImmerse program, we will discuss this interface in more detail. A self-contained, non-cluster program (not using GLUT or Syzygy) could have a flow of five custom-written steps: 1) read tracking/sensors; 2) update-world, based on sensor data; 3) set viewing transform; 4) draw-world; 5) repeat back to step 1.

**Figure 2:**
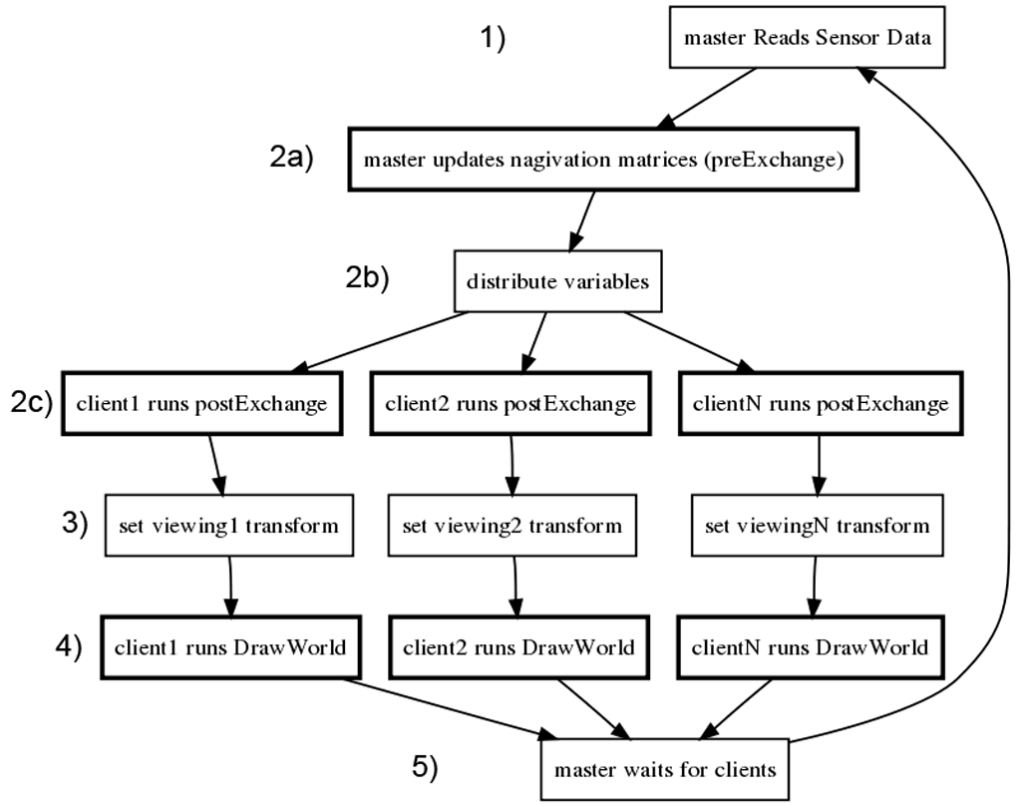
**Flow chart of the KinImmerse logic**. Diagram illustrating the flow of steps in a cycle of the callback-style API for 6-sided display and interactive navigation. Steps in boxes with bold borders are custom written for KinImmerse, while the rest are provided by the Syzygy toolkit.

The Syzygy callback-style API can provide step 1 of reading the sensor data, step 3 of setting the viewing transform based on head position and screen geometry (as specified in a configuration file), and the looping functionality of step 5. To complete this call-back system, the programmer need only write the registered update-world function that decides what objects to modify in the scene (step 2) and the registered draw-world function using OpenGL calls (step 4), for the API to call as needed. Thus we need not be concerned with the specifics of the tracking system used or the screen geometry. In addition, Syzygy provides all the functions necessary to run the application over a cluster of computers. The complete Syzygy version works roughly as diagrammed in Figure 2, where only the functions in bold-outlined boxes are custom coded.

### Data structure design

The internal data design of KinImmerse mimics the hierarchical nature of the kinemage format. As the kinemage file is loaded, a series of containers (groups, subgroups, and lists in kinemage terminology) along with objects (points, lines, spheres, etc.) are created and added to their proper parent objects. There are some differences between the levels in kinemage format (e.g., animation is done on groups, and individual display objects are in lists). These properties are implemented as extra restrictions on the KinImmerse containers also, to ensure that output files produced from the DiVE are compatible with later display on standard single-screen systems using KiNG or Mage.

In addition to the hierarchy of display primitives and their properties (geometry, position, color, name, etc.), the input kinemage file can be used to specify state and scope properties, such as which objects are initially turned on, or the scope of animation or co-centering. The kinemage format also has a cross-cutting system of "masters" that can control display-object visibility with flags that can occur on any object at any of the container levels. Masters will need to be implemented as a special case in KinImmerse, since they are orthogonal to the major kinemage hierarchy. This will be part of a more general future rework of the menuing system.

### Hierachical bounding boxes

Another advantage of using the hierachical internal data representation is ease of integration of axis-aligned bounding boxes. At each item (whether container or object) we compute a bounding box for that item. This is quick to recompute, and in practice is only recomputed for the whole structure after a rotation/translation/scale operation is completed. Having the bounding boxes for all objects, we use a collision detection algorithm which checks to see if the current wand point is inside the container's bounding box, before descending to check if the wand point is inside any of its children. This potentially speeds up the collision detection process by eliminating checks on objects that, as a group, are outside of the wand point.

### File format versus interactive display features

The single-screen kinemage display programs Mage and KiNG both can read, interpret, and write out essentially all aspects defined in the kinemage format. Their basic on-screen functions are very similar, but some of their functionalities for user editing and manipulation differ. Analogously, the new KinImmerse application needs to read, interpret, and write out all basic aspects of the kinemage format, but its interactive display and interaction tools can be quite different, as either required or enabled by the VR environment. These differences, and new user interactions in KinImmerse, are described in the Results and Discussion sections.

### Kinemage construction for the test applications

The kinemage graphics files for demonstration use (ribbons, ball&stick, and space-filling) were produced by the Molikin feature of KiNG, with some minimal on-screen or in-file editing to optimize display in the DiVE. The dot surfaces of all-atom contact analysis were calculated in MolProbity [34]. The NMR structure examples used were the 10-model [PDB:1D3Z] structure of ubiquitin [42], solved from unusually complete NOE and RDC data, the [PDB:1Q2N] Z-domain ensemble [43], and the [PDB:2I5O] polymerase η domain ensemble [44]. The multi-model ensemble kinemages were produced by the MolProbity site's standard procedures for analyzing NMR structures [34]. Approximate starting global superposition of the ensemble models for ubiquitin was done with the docking tools in KiNG, since the models in the deposited 1D3Z PDB file diverge significantly in rotation around the principal direction of the alignment tensor. Color-coded dotted lines to represent NOE data were produced by the NOEdisplay plug-in to KiNG (by Brian Coggins; personal communication).

The kinemage representation of target curves for RDC data was produced by the RDCvis Java program implemented specifically for this project, which can be run independently or as a plug-in to KiNG. In order to generate target curves, RDCvis requires a PDB-format coordinate file and a set of experimental RDC values (on a given type of interatomic vector, in a given alignment medium; RDCs are currently required to be in the format output by CNS). These calculations are done using a method detailed previously [45-47]; briefly, the underlying equation is the following:

$$D_{i}=D_{max}v_{i}^{T}Sv_{i}$$

where *D*_*i *_is the experimental value of the RDC; *D*_*max *_is a constant representing the maximum dipolar coupling based on properties of the atoms, physical constants, and protein dynamics in solution; *v*_*i *_is the internuclear vector; and *S *is the Saupe alignment tensor [48] which is a 3 × 3 order matrix.

From a set of at least five experimental RDCs and coordinates for the atoms of their corresponding bond vectors, RDCvis uses the above equation and singular-value decomposition to calculate an optimal Saupe alignment tensor, whose elements describe the direction and asymmetry of the partial molecular orientation in the experimental system. It then uses the tensor to determine the quartic equation of the RDC curves for each internuclear vector. Each pair of target curves is drawn as polygonal curves that lie on a sphere centered on one of the atoms of the internuclear vector (for instance, the N atom for an NH RDC). The paired curves represent the locus of possible orientations of the vector (and thus possible positions for the H) that are compatible with its experimentally-measured RDC value. To match standard practice in NMR structure determination, RDCvis is usually run to calculate a Saupe tensor separately for each model of the ensemble.

Since the RDC is a measure of interatomic-vector orientation, it is actually undesirable to do a full local superposition of the NMR models using rotational terms. However, combining the purely translational co-centering feature built into KinImmerse with the RDC curve visualizations on a single atom is extremely powerful. It allows users to see instantly how much the local conformations vary and whether some models have internuclear vectors that do not line up correctly on their RDC curves.

### Free Open Source Software

Finally, as we desire to create tools that can benefit and be accessible to as many as possible, we have made the KinImmerse system dependent only on libraries or toolkits that are open-source (e.g. Syzygy, VRPN, KiNG), as well as releasing the application itself under a BSD-style open source license. KinImmerse, KiNG, and RDCvis are available at .

## Results

The KinImmerse system can directly parse nearly all kinemage graphics files and display them on the 6 sides of the DiVE or in other VR systems. Representations include ribbon schematics, stick figures, ball-and-stick, space-filling spheres, electron density contours, dot surfaces, and various abstract notations that use simple graphics primitives (such as symmetry or helix axes, 3D scatterplots of data, or the NMR data representations described in this paper). After user interaction, the modified kinemage file can be written out again, for later use in KinImmerse VR or in Mage or KiNG single-screen or web-based graphics. Loading and saving of files is controlled through a separate Java GUI on the command console.

For general, demonstration-mode molecular displays in the DiVE we have shown ribbon, ball&stick, and space-filling representations of proteins and nucleic acids. Protein/protein or protein/nucleic acid interactions have been shown as polygonal Voronoi surfaces [49] along with all-atom contact dot surfaces [33]. Correction of individual residue conformations in protein crystal structures has been shown by animating between before-and-after kinemages with electron density contours, all-atom contact dots, and local stick figures in a ribbon context. Our major test application for research use – NMR ensembles and data – is described below. For all of the novice users and for nearly all the expert users, it was found anecdotally (from user responses and from watching their head and eye movements) that the KinImmerse display seems to provide significantly better perception of the 3-dimensional relationships than motion plus stereo in single-screen systems. This is especially true for the master user with the wand controller, but also holds for the other viewers.

### User Interface

**Figure 3:**
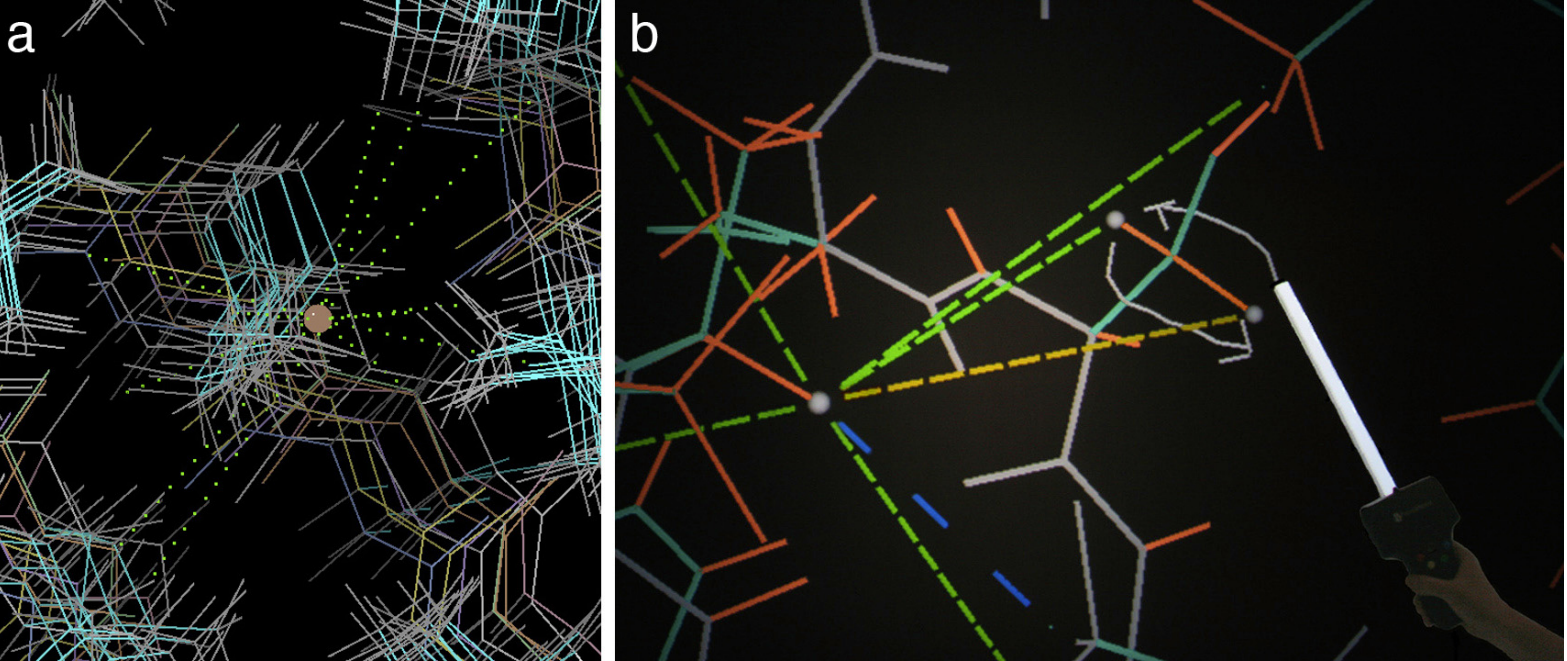
**NOEs for Ile3 Hβ of 1D3Z, in KiNG vs KinImmerse user session**. a) Traditional display of the 10-model 1D3Z ubiquitin ensemble [42] in KiNG, with backbone in white and sidechains cyan. For model 1, the Hβ of Ile3 is highlighted with a brown ball and NOE measurements between it and neighboring atoms are shown as dotted lines. b) Working KinImmerse session in the DiVE, for those same NOEs (dashed lines). All models were co-centered on Ile3 Hβ (gray ball), but only model 1 is shown here for clarity on the flat page; bonds to H atoms are in orange for emphasis. The user has drawn a 3D annotation to indicate that the distances to the two Hβs of Ser65 appear to be reversed. Photographed in the DiVE by JNB, VBC, and DCR.

The interface implements a mix of metaphors from virtual reality and molecular graphics, optimized for domain-specific interactions that enable scientific insight into the 3D structure of biological macromolecules. The dominant schema is that of person-centered control. All users have stereo goggles and can move about freely. The master user wears the head-tracked goggles that control the position and direction of view, navigates and controls mode with the hand-held physical controller, and points its virtual wand to select or grab objects. The InterSense handheld controller acts as a 3D mouse, topped by a central joystick and a crescent of four buttons (red, yellow, green, and blue) in easy thumb reach. The virtual wand appears as a white pointer stick projecting forward from the controller, as shown in Figure 3b.

As well as moving about in the room, the user navigates by a flexible, gaming-style point-to-fly navigation. Pushing on the joystick flies the user through the scene in the direction it is pushed, relative to the direction the handheld controller is pointing. Typically the joystick is pushed forward, to fly toward where the wand is pointing. More push moves faster. For extended work sessions, the user can navigate virtually from a "command chair" with padded feet to protect the DiVE floor, which also allows the use of notes, laptops, or multiple VR interface devices.

The display objects (that is, the molecules) can be manipulated in several different manners. Holding down the yellow button is a "grab" function that locks the graphics to the wand. This enables full 6-degree-of-freedom orientation of the graphics image, since the ultrasonic tracker follows the translational and rotational position and motion of the handheld controller. This feels like grabbing the molecule with your hand and turning it about. While the green button is pressed, pushing the joystick forward or back scales the display larger or smaller. This allows the user to zoom in or out of the display. If an animation pair or sequence is defined in the kinemage (usually to show conformational changes), pressing the red button advances by one step in that sequence.

A KinImmerse menu to show or hide individual elements of the kinemage is presently provided as a fixed menu list along one edge of the left side wall. It uses a direct hit of the virtual wand tip on the menu item to change its state. Being able to turn specific display elements on or off (such as the RDC curves, the multiple models, the H atoms, etc.) is essential for serious production work such as the NMR analyses, and is also very convenient for demonstration or teaching mode.

While the user touches the tip of the virtual wand to a point of interest (an atom in our examples), a bounding box appears around the object and the object's identifying information is shown in one corner of each screen. The content of such information for an atom usually includes molecule, residue type, sequence number, and atom type (plus model number if NMR), but is freely specifiable in the input kinemage file.

Holding down the blue button enables user 3D annotation: drawing a freehand line in 3 dimensions with the tip of the virtual wand as it is moved. The skillful user can write text as well as draw 3D glyphs. These annotation marks become a part of the kinemage, so standard desktop kinemage programs can be used later to view annotations made while immersed in VR space (see below).

Hitting the blue button while holding down the green button (done with a rolling thumb motion) instructs the system to translationally co-center preselected mobile groups (such as NMR models) onto the picked point. This function may be re-assigned to a single button in future, since it has turned out to be centrally important to the RDC analysis. The program identifies which points to co-center by their sharing a common string with the name of the selected point. This illustrates the power of using an authored display descriptor such as the kinemage format. For the NMR examples under study the co-centered points are atoms of the same name in different models of the ensemble, with a set number of characters kept identical in the point names. However, this function could work on any kinemage that has points with well-behaved names. Co-centering is of course a very simple procedure once the relevant atoms have been identified. The novel aspect is identifying this specialized research context in which purely translational co-centering is the correct and useful process, and implementing it with one-click convenience for quickly moving from one locally centered atom to another.

### Local Immersion: Comparing Models with Data in an NMR Ensemble

Our central test of KinImmerse as part of the research process is visualization of NMR structural ensembles and local analysis of the relationships between the NMR experimental data and the models derived from those data. Currently KinImmerse supports representation of NOE distance data as dashed lines and of RDC orientation data as pairs of target curves (see Background for their meaning and Methods for their production).

Figure 3a shows a standard visualization (in KiNG) of the set of NOEs observed for the CβH atom of Ile 3 in the10-model ensemble of the high-accuracy 1D3Z structure of ubiquitin, as deposited. In the DiVE, this system was initially viewed in the prototype Virtools display, where both orientational and translational superposition of models was done in the DiVE by interactive 6-degree-of-freedom docking onto a chosen reference model. We learned that the 1D3Z ensemble is extremely tight once globally superimposed, certainly understandable for models optimized against many NOEs and sets of RDCs in two different media, but not evident in the deposited ensemble (Fig. 3a), which is tightly oriented (by refinement of RDC values) along the principal direction of the alignment tensor but not in rotation around that direction. Interactive translational centering on an atom of interest was found to be extremely valuable in assessing the model-to-data relationships within a local region. Therefore in the current KinImmerse system, global superposition of models is done as a pre-processing step in the rare cases where it is needed, and a new purely-translational local "co-centering" operation was implemented as part of the user interface (see Methods). For the working session illustrated in Figure 3b, the 10 models were all co-centered on the Hβ of Ile 3, but only one model is shown in the figure for clarity on the page.

For studying NOE data in the DiVE, one can locate any restraint violations, color-coded in red (or near-violations in yellow), in the whole ensemble or across one model. In an area of interest, one co-centers the ensemble on a particular H atom for detailed local analysis. 1D3Z has no restraint violations, but we noticed that the relative NOE intensities observed between the central Ile 3 CβH and the two Cβ protons of Ser 65 are reversed relative to the respective interatomic distances in the model. In Figure 3b, the user has drawn two arrows with the 3D annotation tool to record that observation. This type of minor discrepancy could have three quite different origins: a reversed resonance assignment, a different sidechain χ1 conformer for Ser 65, or a quite plausible 1Å measurement uncertainty. Co-centering on Ser 65 Cβ to check its other NOE data, we found that the relative distances for sensitive pairs (from 1Hβ vs 2Hβ, to 65 NH, to 66 NH, and to Phe 45 1 He/2 He) are all neatly reversed by about 1Å as well, which would be unlikely from independent measurement errors. Modeling the other two Ser 65 rotamers in KiNG (not yet implemented in KinImmerse) shows them to have less optimal but not impossible sterics and H-bonding, and to only approximately reverse the NOE distances. The consistent pattern of relative distance reversal is therefore most compatible with reversed assignments of 65 1Hβ and 2Hβ, and could prompt re-examination of the evidence for those assignments. This discrepancy could certainly have been picked up in other graphics systems, or even in lists of distances, but the KinImmerse system is especially suited to quickly test the local consequences for neighboring atoms and data.

### Visualization of RDC data and relationships

**Figure 4:**
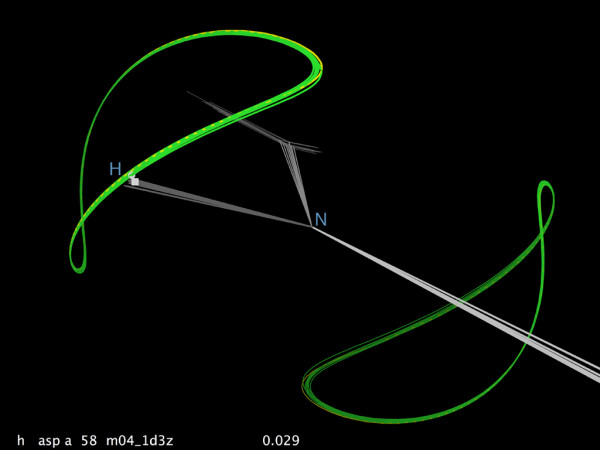
**NH RDC curves, co-centered on the N atom**. An example of RDC target curves (in green) calculated by RDCvis and shown in KinImmerse: backbone NH of Asp38 in 1D3Z, for all 10 models of the ensemble. Co-centered in the DiVE, saved as an output kinemage, and then displayed in KiNG.

Figure 4 shows the pair of target curves for the RDC of a specific NH bond vector in one model of an NMR ensemble, color-coded by model-to-data agreement (see Methods). They lie on a sphere centered on the N atom; if the H atom lies at any point on one of the curves, then its back-calculated RDC would exactly match the observed value – which is very nearly the case for all 10 models in this example. Other possible RDC information can be calculated by RDCvis and viewed in KinImmerse. A curve can be back-calculated from the NH vector orientation in each model, with two outer curve sets showing the results of an RDC either +1 Hz or -1 Hz from the measured value. RDC measurements can be very precise, and in many cases the strips of probable orientation on the sphere are quite narrow. However, the RDC equations are highly non-linear, and in some cases a small change in RDC values can encompass rather large changes in model bond-vector orientation.

In analyzing the 1Q2N ensemble for the Z domain of Staphylococcal protein A [43], one sees that including RDC data improved model-to-data agreement and other quality criteria [50,34] for the backbone compared with the earlier 1SPZ and 2SPZ structures. But the helix 2–3 loop in 1Q2N shows two quite different conformations in nearly equal numbers for residues 37–39, with no restraint violations. We would like to understand how both conformations can fit the RDC data, and if possible to decide whether one of the conformational groupings is wrong or whether the two groups together represent a valid ensemble.

**Figure 5:**
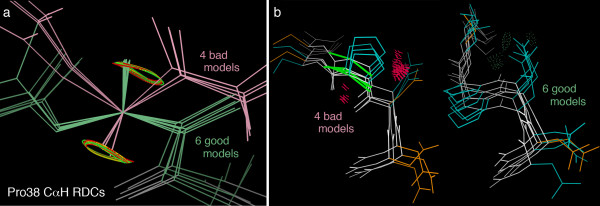
**Evaluating two clusters of loop models by RDC geometry**. Helix-helix loop 36–40 in the 1Q2N Z-domain ensemble [43] shows two quite different conformational clusters of models, in spite of the fact that all 10 models match the experimental RDC data that measures orientation. a) Pro38 CαH bond vectors for the two model clusters point in nearly opposite directions, matching opposite limbs of the paired RDC target curves. b) Quality comparison of the two model clusters, from the MolProbity structure-validation site [34]: the 4 bad models have Ramachandran outliers (green), many steric clashes (hot pink spikes) and sidechain rotamer outliers (gold), while the 6 good models have no clashes or Ramachandran outliers, few bad rotamers, and several hydrogen bonds (lenses of green dots). One can conclude that the RDC match for the 4-model conformation is a coincidence, and those models are incorrect.

Figure 5a shows this loop region of the 1Q2N ensemble backbone in KinImmerse co-centered on the Cα atom of Pro 38, with the CαH RDC curves on each model. The two clusters of different loop conformations are colored pink and green (done in KiNG). Residue 38 is a proline which therefore has no NH, and the 37 and 39 CαH RDCs were not observed, a level of data incompleteness not uncommon in loops. The ensemble sampling identified unique conformational clusters for residues 36 and 40, anchoring the loop ends at the C-cap and N-cap of the two helices it joins. The largest conformational difference is at Pro 38, and it can be seen in Fig. 5a that the Cα-CαH bond vectors point in nearly opposite directions for the two model clusters and that their CαH atoms lie on opposite limbs of the RDC target curves. Each individual model of the two conformational clusters was built with a target of matching the CαH atom position to some point on the two-limbed RDC curve. However, the overall ensemble of vectors for this Cα-CαH bond would not produce the measured RDC value if they actually had such a diverse mix of orientations. Therefore this coincidence of good matches on both limbs of the RDC curve must be a modeling artifact, and one of the clusters is presumably incorrect.

Other local NMR data in the 37–39 loop is relatively sparse and has already been taken into account in the structure-solution process, but we can obtain an independent assessment of the clusters from the MolProbity structure validation site. Figure 5b shows the multi-criterion MolProbity kinemage [34] for this region of the 1Q2N ensemble, with Ramachandran outliers marked in bright green, sidechain rotamer outliers in gold, all-atom steric clashes as clusters of hot pink spikes, and hydrogen bonds (H-bonds) as lenses of pale green dots. The conformational cluster of models 2, 3, 7, and 8 has many clashes and rotamer outliers and no H-bonds, while the six models in the other conformational cluster have no clashes, are nearly free of outliers and make several H-bonds. The conclusion, therefore, is that only the 6-model cluster represents a valid conformation for this loop and the Pro 38 CαH vector should match the upper branch of the RDC target curve in Fig. 5a.

In addition to obtaining specific new information about the model-to-data relationships in NMR structural ensembles for two different proteins, we were convinced of the value of the co-centering operation pioneered in KinImmerse, which has now also been implemented as a tool in the traditional KiNG display system. Such propagation of new visualization and interface ideas out into non-VR software is another kind of benefit from exploratory visualization research in systems such as the DiVE.

## Discussion

### Demo mode

**Figure 6:**
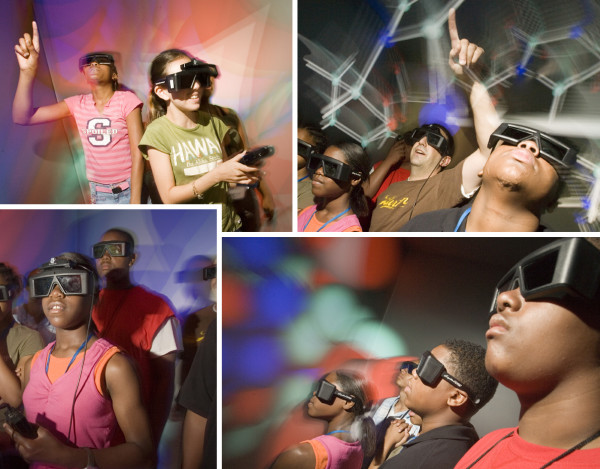
**High school students experiencing a molecule in the DiVE**. Students in the Howard Hughes phage-hunters summer program locating structural features of a DNA double helix in the DiVE. These demo sessions used the VirTools prototype of the VR kinemage display system (see Methods). Photography by Chris Hildreth, Director of Duke University Photography.

KinImmerse (and the earlier prototype Virtools system) was immediately successful in demonstration ("demo") mode, engaging and exciting viewers from all levels of sophistication, who enjoyed flying through the major and minor groove of DNA or having the β-barrel ribbon of a membrane-pore protein pulled down over their heads. Pharmacology and engineering classes were motivated by having their projects end up as kinemages in the DiVE. In a Howard Hughes "Phage Hunting" summer program for at-risk high school students, the DiVE session (shown in Figure 6) was their highest-rated section of the program. Such demo and educational uses are certainly not new to this application and are not its major purpose, but they are a very worthwhile side benefit.

### Differences from single-screen molecular graphics or from most VR

KinImmerse uses a combination of the conventions from single-screen molecular graphics and usual VR applications, and thus differs in some ways from either. Navigation and viewpoint are quite unlike the window-into-a-box of traditional molecular graphics, where one centers on a point by name, by preset views, or by picking and zooming, and where stereo and display-object motion by dragging give a good perception of depth, but the viewer is always on the outside. In VR the viewer is inside the world of the display, and walking or 3D flying to a point of interest is the dominant metaphor. In KinImmerse the user can move about freely in the room/molecule or can push on the joystick of the handheld controller and fly (virtually) in that direction. Up to 5 or 6 users can be inside the DiVE together, all with stereo goggles but only one with the head-tracked master goggles and the hand-held controller. The controller has a virtual wand extension whose tip can directly select points in 3D or grab and manipulate display objects, giving the user very active and tactile control.

On the other hand, KinImmerse supports line and dot graphics primitives, in addition to the triangulated surfaces that are the assumed primitives in nearly all VR displays. The information text (shown in a corner when a point is selected) goes beyond purely visual presentation into more specific and quantitative interactions with the displayed molecules, while the command-chair mode makes extended work sessions feasible. As far as we know, freehand 3D annotation, one-click co-centering, and local display of RDC curves are all novel functionalities in either single-screen or VR applications. RDC visualization and co-centering provide a completely new type of analysis, while the VR environment is found anecdotally to improve manipulation and perception of the 3D relationships. We believe this unusual combination of immersive context and detailed control should enable enhanced types of molecular research.

### Future Directions

KinImmerse now provides a functional and self-contained system for doing macromolecular research in immersive VR, but we plan specific improvements in several aspects that can further enable production research uses. The interface features can be reorganized and broadened to provide a more graceful menu system, even easier co-centering, and the option for two-handed controls. Display of information text in the DiVE corners is very effective and unobtrusive, and that mode could also support distance and other measurements. A way to pre-specify a startup view or location would strengthen multi-session use. We plan to further optimize the lighting model for different molecular representations. We will explore ways to represent other NMR data types, such as dihedral-angle restraints and order parameters. On the hardware side, a new computer cluster will soon be installed, giving higher fill rate, increased memory, and programmable shaders.

Most importantly, KinImmerse will now allow NMR domain experts to explore the use of local immersion, co-centering, and experimental RDC data display for research analysis of NMR structural ensembles. We will encourage such use in the DiVE, both for its scientific value and to learn what aspects of the research use are most enhanced by VR.

## Conclusion

Combining the DiVE immersive VR hardware, the Syzygy VR toolkit, and the kinemage graphics format has enabled development of the open-source KinImmerse software system for immersive molecular graphics in the service of production research uses. KinImmerse takes advantage of flexibly customized input from the KiNG single-screen software, including a new visualization of RDC target curves on the NMR models. It then contributes immersion, navigation, and manipulations from the VR tradition; identification, measurements, and output from the single-screen tradition; and introduces new interactive research functions of 3D annotation and co-centering. The initial research application explores the study of NMR experimental data, especially RDC's, in context of one-click local co-centering of models in the NMR structural ensemble. This test case sparked new scientific insights now being pursued. Long-term success would constitute having NMR structural biologists using these tools routinely to help improve the quality of their structures or their understanding of them.

## Competing interests

The authors declare that they have no competing interests.

## Authors' contributions

JNB, JSR, DCR and RB conceived the project. RB provided the DiVE facility. DJZ programmed KinImmerse. JNB wrote the Virtools prototype with help from ECV. VBC wrote RDCvis, and IWD, JNB and ECV programmed related utilities. JNB, VBC, JSR, DCR, and DJZ devised, performed, and evaluated the research usage tests. JNB, DJZ, and DCR conducted the demo and teaching sessions. JSR, JNB, DJZ, VBC, and DCR wrote the paper, which all authors have read and approved.

## References

[B1] Richardson DC, Richardson JS (2002). Teaching molecular 3-D literacy. Biochem Molec Biol Educ.

[B2] Katz L, Levinthal C (1972). Interactive computer graphics and representation of complex biological structures. Ann Rev Biophys Bioengin.

[B3] Porter TK (1978). Spherical shading. Computer Graphics.

[B4] Britton EG, Lipscomb JL, Pique ME (1978). Making nested rotations convenient for the user. Computer Graphics.

[B5] Jones TA (1978). A graphics model building and refinement system for macromolecules. J Applied Crystallogr.

[B6] Connolly ML (1993). Solvent-accessible surfaces of proteins and nucleic acids. Science.

[B7] Richardson JS (1981). The Anatomy and Taxonomy of Protein Structure. Adv Prot Chem.

[B8] Carson WM, Bugg CE (1986). Algorithm for ribbon models of proteins. J Molec Graphics.

[B9] Richardson DC, Richardson JS (1992). The kinemage: a tool for scientific illustration. Protein Science.

[B10] Sayle R, Milner-White EJ (1995). RASMOL: Biomolecular graphics for all. Trends Biochem Sci.

[B11] DeLano WL (2002). The PyMOL molecular graphics system.

[B12] Guex N, Peitsch MC (1997). SWISS-MODEL and the Swiss-PdbViewer: An environment for comparative protein modeling. Electrophoresis.

[B13] Lovell SC, Davis IW, Arendall WB, de Bakker PIW, Word JM, Prisant MG, Richardson JS, Richardson DC (2003). Structure validation by Cα geometry: φ, ψ and Cβ deviation. Proteins.

[B14] Pettersen EF, Goddard TD, Huang CC, Couch GS, Greenblatt DM, Meng EC, Ferrin TE (2004). UCSF Chimera – A visualization system for exploratory research and analysis. J Comput Chem.

[B15] Koradi R, Billeter M, Wüthrich K (1996). MOLMOL: a program for display and analysis of macromolecular structures. J Mol Graphics.

[B16] Emsley P, Cowtan K (2004). Coot: model-building tools for molecular graphics. Acta Crystallogr.

[B17] Humphrey W, Dalke A, Schulten K (1996). VMD – visual molecular dynamics. J Molec Graphics.

[B18] Sherman WR, Craig AB (2002). Understanding Virtual Reality: Interface, Application, and Design.

[B19] Arthur K, Preston T, Taylor RM, Brooks FP, Whitton MC, Wright WV (1998). The PIT: Design, Implementation, and Next Steps. Proceedings of the 2nd International Immersive Projection Technology Workshop.

[B20] Surles MC, Richardson JS, Richardson DC, Brooks FP (1994). Sculpting proteins interactively: Continual energy minimization embedded in a graphical modeling system. Protein Sci.

[B21] Taylor RM, Robinett W, Chi VL, Brooks FP, Wright WV, Williams RS, Snyder EJ (1993). The Nanomanipulator: A Virtual-Reality Interface for a Scanning Tunneling Microscope. ACM SIGGRAPH Proc.

[B22] Fisher J, Cummings J, Desai KV, Vicci L, Wilde B, Keller K, Weigle C, Bishop G, Taylor RM, Davis CW, Boucher R, O'Brien ET, Superfine R (2005). Three-dimensional force microscope: A nanometric optical tracking and magnetic manipulation system for the biomedical sciences. Rev Scientific Instruments.

[B23] Brady R, Pixton J, Baxter G, Moran P, Potter CS, Carragher B, Belmont A (1995). Crumbs: A virtual tracking tool for biological imaging. Proceedings of the 1995 Biomedical Visualization Conference.

[B24] Cruz-Neira C, Sandin D, DeFanti T (1993). Surround-screen projection-based virtual reality: The design and implementation of the CAVE. ACM SIGGRAPH Proc.

[B25] Sherman W FreeVR Home Page. http://www.freevr.org.

[B26] Stone J VMD Publications. http://www.ks.uiuc.edu/Research/vmd/publications/cave2001.pdf.

[B27] Ferey N, Delalande O, Grasseau G, Baaden M (2008). A VR framework for interacting with molecular simulations. Proceedings of the ACM Symposium on Virtual Reality Software & Technology.

[B28] Moritz E, Meyer J (2004). Interactive protein structure visualization using virtual reality. Proceedings of the 4th IEEE Symposium on Bioinformatics and Bioengineering.

[B29] PDB and CalIT2 (2006). PDB in a CAVE: Virtual reality environment highlights PDB structures. PDB Newsletter spring.

[B30] Jean Goldwurm 3D Visualization Theater. http://www.weizmann.ac.il/ISPC/3dtheater/index.html.

[B31] Berman HM, Westbrook J, Feng Z, Gilliland G, Bhat TN, Weissig H, Shindyalov IN, Bourne PE (2000). The Protein Data Bank. Nucleic Acids Res.

[B32] Cavanagh J, Fairbrother WJ, Palmer AG, Rance M, Skelton NJ (2006). Protein NMR Spectroscopy: Principles and Practice.

[B33] Word JM, Lovell SC, LaBean TH, Zalis ME, Presley BK, Richardson JS, Richardson DC (1999). Visualizing and Quantitating Molecular Goodness-of-Fit: Small-probe Contact Dots with Explicit Hydrogen Atoms. J Mol Biol.

[B34] Davis IW, Leaver-Fay A, Chen VB, Block JN, Kapral GJ, Wang X, Murray LW, Arendall WB, Snoeyink J, Richardson JS, Richardson DC (2007). MolProbity: All-atom contacts and structure validation for proteins and nucleic acids. Nucleic Acids Res.

[B35] Arendall WB, Tempel W, Richardson JS, Zhou W, Wang S, Davis IW, Liu Z-J, Rose JP, Carson WM, Luo M, Richardson DC, Wang B-C (2005). A test of enhancing model accuracy in high-throughput crystallography. J Struct Funct Genomics.

[B36] Headd JJ, Immormino RM, Keedy DA, Emsley P, Richardson DC, Richardson JS (2008). AutoFix for backward-fit sidechains: Using MolProbity and real-space refinement to put misfits in their place. J Struct Funct Genomics.

[B37] Richardson DC, Richardson JS, Rossmann M, Arnold E (2001). MAGE, PROBE, and Kinemages. Chapter 2528 in IUCr's International Tables for Crystallography, Volume F: Crystallography of Biological Macromolecules.

[B38] Schaeffer B, Goudeseune C (2003). Syzygy: Native PC cluster VR. Technical report from the Integrated Systems Laboratory.

[B39] Taylor RM, Hudson TC, Seeger A, Weber H, Juliano J, Helser AT (2001). VRPN: A device-independent, network-transparent VR peripheral system. Proceedings of the ACM Symposium on Virtual Reality Software & Technology: 2008.

[B40] Mechdyne. http://www.mechdyne.com/integratedSolutions/software/products/trackd/trackd.htm.

[B41] Kilgard MJ (1996). The OpenGL utility toolkit (GLUT) programming interface: API version 3.

[B42] Cornilescu G, Marquardt JL, Ottiger M, Bax A (1998). Validation of protein structure from anisotropic carbonyl chemical shifts in a dilute liquid crystalline phase. J Amer Chem Soc.

[B43] Zheng D, Aramini JM, Montelione GT (2004). Validation of helical tilt angles in the solution NMR structure of the Z domain of Staphylococcal protein A by combined analysis of residual dipolar coupling and NOE data. Protein Sci.

[B44] Bomar MG, Pai MT, Tzeng SR, Li SS, Zhou P (2007). Structure of the ubiquitin-binding zinc finger domain of human DNA Y-polymerase eta. Embo Reports.

[B45] Losonczi JA, Andrec M, Fischer MWF, Prestegard JH (1999). Order matrix analysis of residual dipolar couplings using singular value decomposition. J Magn Reson.

[B46] Yan AK, Langmead CJ, Donald BR (2005). A probability-based similarity measure for Saupe alignment tensors with applications to residual dipolar couplings in NMR structural biology. Internat J Robotics Res.

[B47] Wedemeyer WJ, Rohl CA, Scheraga HA (2002). Exact solutions for chemical bond orientations from residual dipolar couplings. J Biomolec NMR.

[B48] Saupe A (1968). Recent results in the field of liquid crystals. Angewandte Chemie.

[B49] Ban YA, Edelsbrunner H, Rudolph J (2004). Interface surfaces for protein-protein complexes. Proceedings of the 8th Annual International Conference on Research in Computational Molecular Biology.

[B50] Huang YJ, Powers R, Montelione GT (2005). Protein NMR recall, precision, and F-measure scores (RPF scores): Structure quality assessment measures based on information retrieval statistics. J Am Chem Soc.

